# Designing Emotion-Integrated Visualizations for Kidney Function Self-Management: User-Centered Design Study With Youth Transplant Recipients and Caregivers

**DOI:** 10.2196/80481

**Published:** 2026-06-15

**Authors:** Chelsea Ng, Julia C Dunbar, Lily Jeffs, Sanaa Syed, Nathan Pan, Hyeyoung Ryu, Jodi Smith, Jaime Snyder, Wanda Pratt, Ari Pollack

**Affiliations:** 1 School of Medicine University of Washington Seattle, WA United States; 2 Information School University of Washington Seattle, WA United States; 3 Boston University Boston, MA United States; 4 Department of Pediatrics University of Washington Seattle, WA United States; 5 Division of Nephrology Seattle Children's Hospital Seattle, WA United States

**Keywords:** adolescents, caregivers, digital health, information visualization, kidney transplant, self-management, user-centered design, young adult

## Abstract

**Background:**

Understanding complex health information, such as kidney function values (eg, creatinine), is important for youth kidney transplant recipients and caregivers to effectively engage and participate in their care. Information visualizations, such as visual analogies, highlight the similarities between 2 different ideas through visual means and can support understanding of abstract data to facilitate self-management. The study was motivated by the persistent challenge that youth and caregivers face in interpreting complex clinical data, which often remains unactionable and disconnected from their practical information needs.

**Objective:**

This study aims to design and evaluate a novel visualization to support the information needs of youth kidney transplant recipients and caregivers, with the goal of increasing actionable and accessible knowledge for self-management.

**Methods:**

We conducted 2 studies with youth kidney transplant recipients and their caregivers: study 1 identifies visualization elements that support patient and caregiver kidney function information needs, and study 2 evaluates a novel patient-facing kidney function visualization informed by results from study 1. In study 1, participants drew representations of their kidney function over time. These findings led to the development of the novel kidney bean visual. In study 2, participants interacted with the personalized (incorporating electronic health record data) kidney bean visual component for 1 week, then completed a feedback session exploring how the novel visual facilitated retrospective reflection of their health status and the specific behaviors influencing their renal function. We conducted qualitative content analysis on all interviews. All sessions and interviews were recorded, transcribed, and analyzed using content analysis (study 1), thematic analysis, and affinity diagramming (study 2).

**Results:**

In study 1 (n=30), youth and caregivers consistently described the difficulty of understanding their creatinine results and related medical terminology, with the majority using emotions to represent their kidney function. Therefore, our kidney bean visual, a visual analogy, incorporated emotions and colors correlating with their creatinine levels and overall kidney function. In study 2 (n=23), we found that for many participants, the kidney bean visual (1) mirrored and validated their emotions, prompting reflections on their kidney function contextualized by their lived experiences; (2) served as a progress check; and (3) acted as a motivator for healthy kidney care habits.

**Conclusions:**

Kidney function laboratory values, although seemingly straightforward in their intended purpose and use, are more complex from the patient and family perspective, as they also closely align with youth kidney transplant recipients’ and their families’ emotions. This study demonstrates the value of designing visualizations that capture these complexities, which can create opportunities for increased understanding of health information and self-reflection to support self-management.

## Introduction

Adolescents and young adults (AYAs) with end-stage kidney disease are at higher risk for self-management challenges and poor transplant outcomes compared with both older and younger populations [1]. A positive outcome following a kidney transplant depends both on the transplant team and the lifelong commitment and engagement of the impacted individual [2]. AYA kidney transplant recipients and their caregivers are expected to take an active role in their health posttransplant in the form of engagement and self-management, which has been shown to improve health outcomes, including increased survival of the transplanted organ, and enhanced quality of life [3,4]. For those living with a chronic illness, self-management requires that they (1) medically manage their condition, (2) maintain or adopt new behaviors to help achieve their goals, and (3) cope with the emotional challenges of living with a chronic condition [5].

To support the high frequency of decision-making required to effectively self-manage one’s health after receiving a kidney transplant, recipients and their caregivers require accessible and understandable health information to achieve their goals. Shared decision-making, in which clinicians and patients work together to make informed choices based on the best available evidence, ensures that care decisions align with patients’ values and needs while respecting their autonomy [6]. However, for patients and caregivers to meaningfully participate in shared decision-making, they need tailored information to support their needs and apply new knowledge to their own health decisions [7]. This support helps them recognize the value of their contributions, fostering a more informed and collaborative decision-making process, resulting in effective self-management.

In the field of pediatric transplantation, the lack of accessible, longitudinal data visualization drives illness uncertainty, a cognitive state elicited when an individual has difficulty understanding the meaning of an illness-related event because health outcomes are unpredictable, illness symptoms of events are ambiguous, or there is a lack of information about the illness or treatment [8]. While clinical conversations with this population focus heavily on creatinine as a key contributor to kidney function, limited research has examined the depth of youth and caregiver understanding of this concept or the effectiveness of existing materials in translating this relationship into actionable self-management strategies. When this complex data—data that demand interpretation across clinical, behavioral, and lived experience dimensions—are presented without clear, accessible context, they lead to a limited information environment that is linked to increased parental distress, lower patient self-management, and ultimately, poorer long-term clinical outcomes [9].

One promising approach to facilitating this understanding is the use of visualizations. Defined as the process of transforming data, information, and knowledge into visual form, visualizations can help users interpret complex information by leveraging the human ability to visually recognize patterns (eg, differences in shapes, sizes, colors, and spatial relationships) to assign meaning [10,11]. By making abstract health data more accessible, visualizations can support patients in contextualizing their own health information, regardless of their level of health literacy or numeracy [11,12]. Further supporting their utility, information visualizations can also be integrated into widely used digital platforms such as mobile apps, websites, or even patient portals, making them a convenient and scalable solution for enhancing patient comprehension in shared decision-making. Despite their potential, patient-facing health visualizations remain underdeveloped, especially for youth populations, compared with those designed for health care providers. A systematic review conducted by Turchioe et al [13] found that a significantly larger number of articles focused on health care provider–facing visualizations compared with patient-facing visualizations [13]. Among the patient-facing visualizations that do exist, prior work has established that presentation format significantly shapes patients’ affective (ie, how results make them feel) and cognitive perception of laboratory results, with evidence favoring visual formats that include contextual reference ranges over numeric displays alone [13,14]. However, nearly all this research has examined single-time-point results, and how youth interpret longitudinal trends in their laboratory values, which are critical to self-management of chronic conditions such as kidney transplant, remains poorly understood. This highlights a broader gap in tools designed to support patients in understanding their personal health information and engaging in informed decision-making. Recognizing this, we explored how a novel information visualization might facilitate the translation of complex clinical data, specifically longitudinal kidney function laboratory values, into meaningful, reflective understanding for AYA kidney transplant recipients, supporting them in better understanding of their health and engaging more actively in their care.

We addressed this through 2 sequential studies, first designing and then evaluating a novel visualization of kidney function laboratory values:

- Study 1: what visualization elements address the information needs and illness uncertainty of AYA kidney transplant recipients and their caregivers to support the reflection necessary for active engagement in their care?
- Study 2: how does a novel visualization incorporating these elements facilitate the translation of complex clinical data into meaningful, reflective understanding that supports youth with chronic kidney disease (CKD) in engaging more actively in their health?

Together, these studies identify the information needs of AYA kidney transplant recipients and their caregivers, demonstrate how a novel visualization can support meaningful reflection on complex health data, and offer design recommendations to guide future development of patient-facing health visualizations for youth with chronic conditions.

## Methods

### Overview

**Figure 1:**
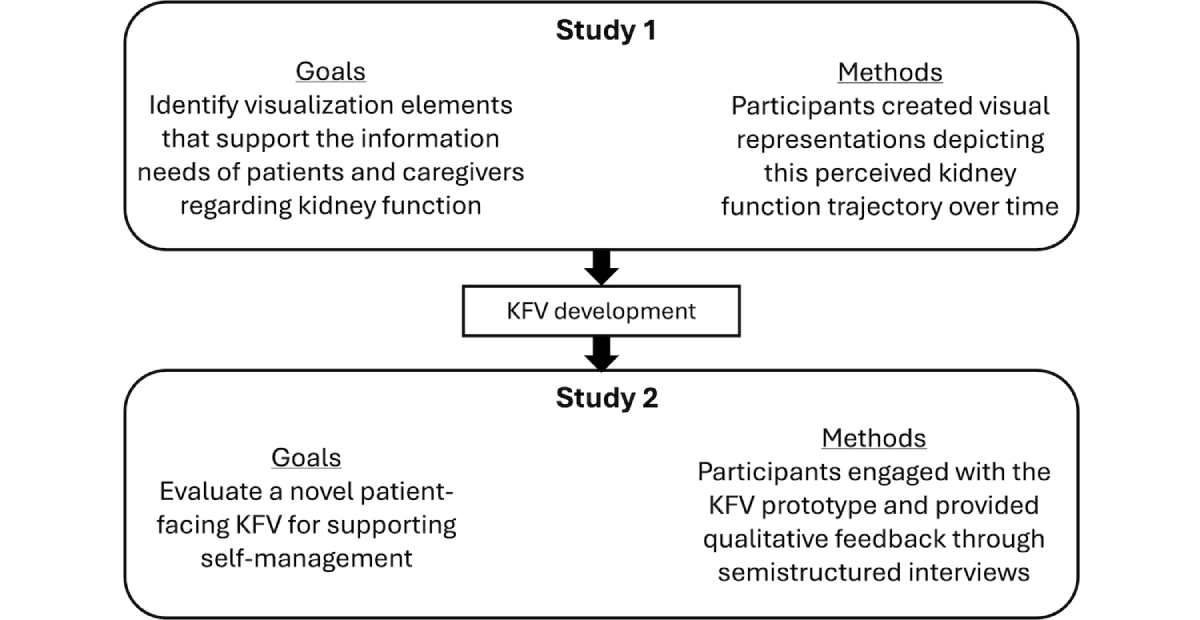
Flowchart of the research phase. KFV: kidney function visualization.

This work is part of a larger multipart study that aimed to design a multifaceted app prototype supporting the information needs of AYAs who have received a kidney transplant and their caregivers. In this study, we present 2 studies (Figure 1) that focus on the development and evaluation of a novel patient-facing visualization. This visualization was incorporated into a multifaced app prototype to enhance understanding of kidney function and support patients in self-management. Study 1 used a series of user-centered design sessions to inform the design and development of a patient-facing visualization of key metrics related to kidney function. The research team took the findings from study 1 and then designed the visualization for study 2, which focused on piloting and evaluating the visualization through a user feedback session.

Focus groups and interviews were facilitated by 3 researchers (CN, JCD, and AP) with prior experience in qualitative inquiry. The team represented a range of academic and professional tiers, including a clinical research coordinator, a PhD candidate in her final year, and an associate professor/nephrologist. Drawing prompts and semistructured interview guides were developed by the authors and pilot tested for clarity and age appropriateness among a small sample of youth aged 12-21 years, including those with and without CKD.

### Recruitment

Participants from study 1 were recruited from Seattle Children’s Hospital (SCH), The Johns Hopkins Hospital, and the Improving Renal Outcomes Collaborative Community Engagement Workgroup, a learning health network designed to improve the lives of children and AYAs who have had a kidney transplant [15]. Participants from study 2 were recruited only from SCH. A single-site approach was adopted because the SCH population was sufficient to meet study objectives.

Recruitment at SCH and Johns Hopkins Hospital was done via convenience sampling by identifying patients (and their caregivers) from a transplant clinic list (nephrology and transplant teams maintain a list of patients who have received a kidney transplant and are currently receiving care). Patients had to meet the following inclusion criteria to participate in either study: aged 10-21 years, had received a kidney transplant at least 6 months earlier, had access to the internet, and were English-speaking (both studies) or Spanish-speaking (study 1 only). Participants were initially contacted for recruitment either in person during their clinic visit, where they were given a recruitment flyer, or remotely via phone call or email. Initial recruitment via email or phone call was used in cases in which email communication already occurred clinically and a patient’s email address was already known to the study team. Improving Renal Outcomes Collaborative participants (study 1) were invited to participate through distribution of a recruitment flyer sent via existing email distribution lists.

During the informed consent process, participants were briefed on the study objectives, and researchers addressed all participant inquiries to ensure transparency. To establish rapport and context, 1 investigator (JD) shared a personal history of kidney disease, and another (AP) disclosed their role as a nephrology provider, although it was ensured that none of AP’s own patients were recruited for the study.

### Procedures

#### Study 1: User Information Requirements and Development of Kidney Function Visualization Interface

We conducted 8 separate 90-minute design sessions using the video conferencing platform Zoom [16], with 4 sessions exclusively with caregivers and 4 sessions exclusively with youth. These sessions were scheduled independently and organized by the youths’ age ranges (eg, aged 10-12 years, aged 13-17 years, and aged 18-21 years) and preferred language (English or Spanish). To facilitate small-group interaction within each 90-minute meeting, participants were placed into separate breakout rooms of 3-5 individuals, each led by a research facilitator. At the conclusion of each design session, all participants returned to the main Zoom room to present and discuss the materials they created within their respective breakout rooms. Participants received a US $25 Amazon electronic gift card for their participation.

To equip participants for their design session, the research team emailed them a design session agenda and mailed them a physical design session toolkit before their session. Design toolkits included the following materials: printouts of the agenda and task cards, colored markers, example visualizations, additional drawing and writing paper for tasks, and emoji stickers representing various emotions, objects, animals, foods, activities, travel locations, and more. Initially, in the design sessions, we asked participants to create a line graph of their creatinine, a waste product filtered by the kidneys commonly used as an indicator of kidney function, using a printout with unlabeled x- and y-axes from their design toolkit (Textbox 1). Participants were asked to create a line graph because this is how kidney function over time is typically presented in the clinic. We also asked them to refer to a printout of an example line graph to aid in their drawings (Figure S1 in Multimedia Appendix 1).

We considered graphs to be successful or correct based on the following criteria: (1) the graph aligned with the concepts that we asked participants to draw, (2) the graph had x- and y- axes with appropriate increments and levels based on the prompt, and (3) the participant’s description correlated with what they drew. Further, 2 primary researchers independently graded each submission and met to resolve any discrepancies until a final consensus was reached. We did not review participants’ data to verify accuracy.

However, after we completed the design sessions with 4 groups of participants (2 youth groups and 2 caregiver groups), we found that youth and caregivers struggled with the task of representing their data on a graph. Therefore, for the remaining 4 participant groups (2 youth groups and 2 caregiver groups), we broadened the prompt and made it easier to understand by removing the requirement to use creatinine specifically or to represent their data on a line graph. Instead, we invited them to draw any image, figure, or visual that represented their kidney function over time (Textbox 1). Drawings were completed individually by all participants and then presented to the others in their session.

**Study 1 design session: original prompt and new prompt.**

**Original prompt—task 2, part 1**

For this task you will have 10 minutes to use your materials in your design toolkit to create 2 drawings. We would specifically like you to make sure to use your emoji stickers within your line graph drawings.

- The first drawing should be a line graph of what you think a perfect creatinine level should look like over time.
- The second drawing should be a line graph of what your creatinine levels have looked like since your transplant.

**New prompt—task 2 part 1**

For this task you will have 5-10 minutes to use your design toolkit to create a drawing. We encourage you to use all parts of your toolkit, such as the colored markers and emoji stickers. Feel free to use any household items you have as well.

- The drawing should be an image, figure, or other representation of your kidney function over time. You will need to include specific elements in your image or figure; please see the drawing paper in your toolkit for further instructions about these elements.

#### Bridging Study 1 and Study 2—Visualization and Tool Design

**Figure 2:**
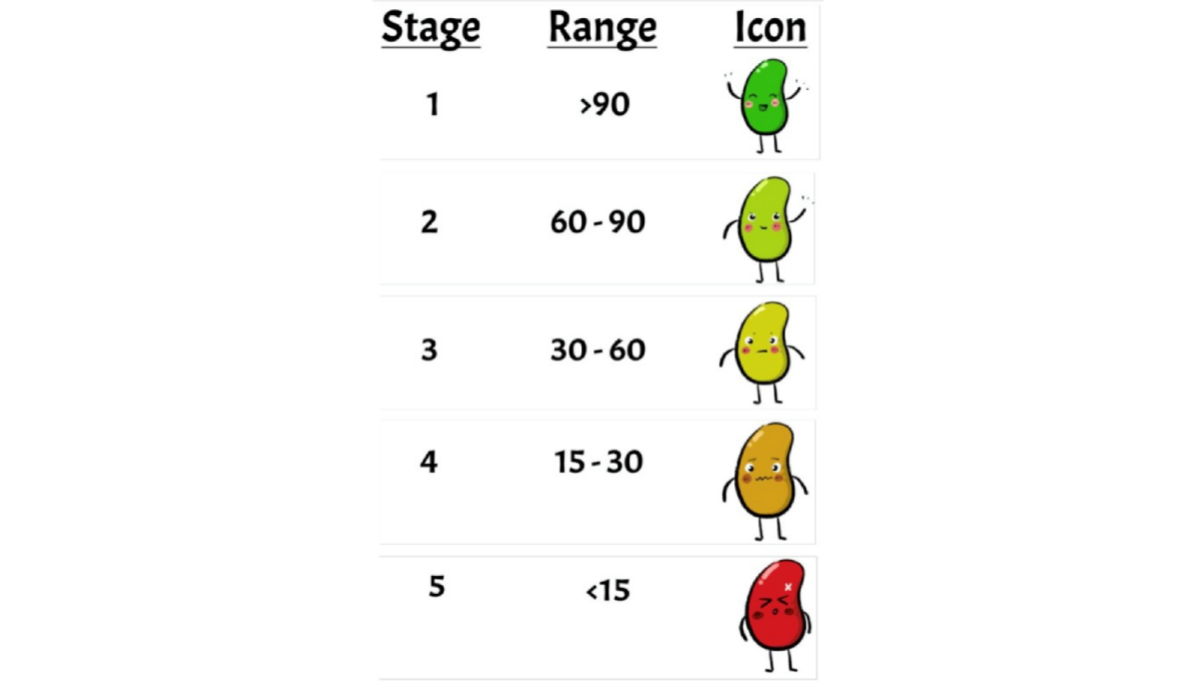
Key of chronic kidney disease stage and glomerular filtration rate value with corresponding kidney bean visual that only researchers had access to.

Based on the findings from study 1, we created the kidney bean visual, which is a cartoon kidney bean with various facial emotions and colors aligned to different stages of kidney function (Figure 2 [17]). This visualization was designed to be a key feature of a larger mobile app prototype, the My Kidney TREK (Thinking, Reflecting, and Empowering for Kidney Transplant Recipients) app*.* This app included 3 main components highlighting kidney function, medication adherence, and storytelling to support the user’s reflection and health engagement in their kidney transplant journey. In this study, we present our findings only on the design of the kidney bean visual feature and accompanying kidney function component.

**Figure 3:**
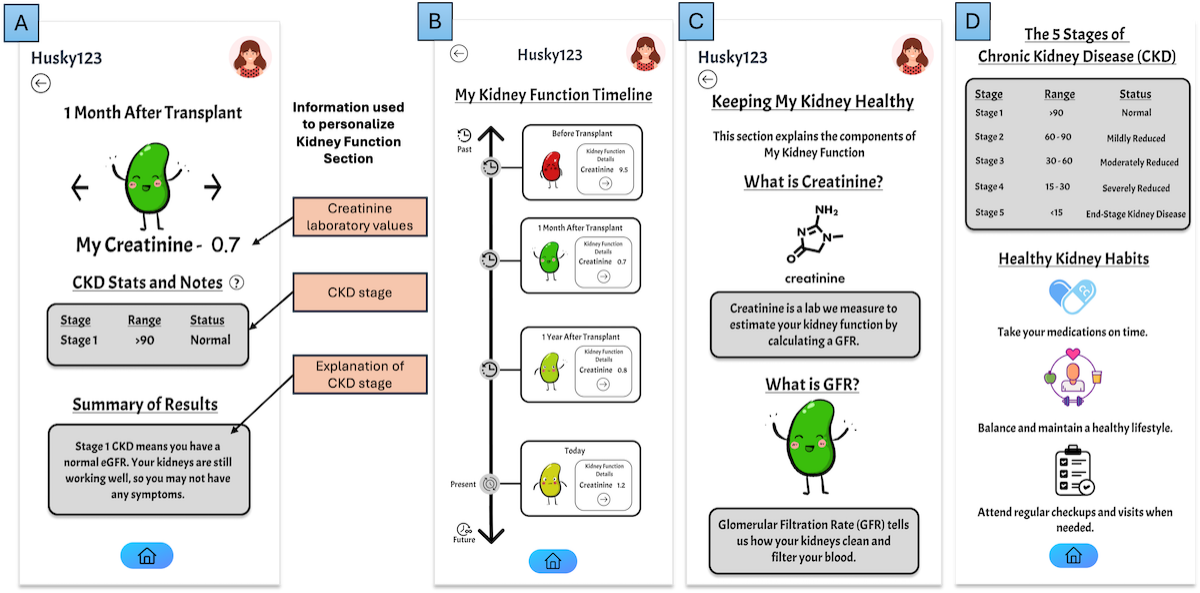
Kidney function component. (A) Individual results page that includes personalized participant data (from the electronic health record), (B) timeline page that includes the kidney bean visual and corresponding creatinine values at 4 different time points, (C) definitions of creatinine and glomerular filtration rate (GFR), and (D) description of chronic kidney disease (CKD) stages and a list of healthy kidney habits.

The prototype was created in Figma, a collaborative web app for interface design [18]. In our novel kidney bean visual, we represent kidney function values through the personification of the kidney organ, with colors and emotions that correspond with creatinine and glomerular filtration rate (GFR) value (ie, green, happy bean for lower creatinine values and better kidney function; Figure 2). The aim of the personification of the kidney bean visual was to act as a cognitive representation of the abstract concept of kidney function values by basing it on the physical functioning of the kidney and the emotions associated with it. The kidney function section was designed to be personalized with creatinine laboratory values retrieved from the user’s medical chart, the corresponding CKD stage, and an explanation of the stage (Figure 3A). Participant’s CKD stage was determined by a pediatric nephrologist on the research team. The stage of CKD refers to how functional the kidneys are, with higher stages corresponding to lower function. CKD stage is determined by calculating the estimated GFR from an individual’s creatinine level. The GFR indicates how well the kidneys are filtering waste from the blood [17]. Creatinine values were also displayed alongside the kidney bean visual (Figures 3A and B). Values from 4 different time points were displayed: (1) before kidney transplant, (2) 1 month after kidney transplant, (3) 1 year after kidney transplant, and (4) the most recent laboratory value at the time of the study. We chose multiple time points to represent the concept of a journey (Figure 3B). Additionally, definitions of creatinine and GFR, as well as healthy kidney habits, were included (Figures 3C and D).

#### Study 2: Piloting the Kidney Bean Visual and Accompanying Component

We piloted the kidney bean visual and corresponding kidney function component of the My Kidney TREK app prototype with a cohort of AYAs and caregiver participants. Further, 2 sessions were conducted: an initial guided walkthrough session to introduce the prototype, followed by a user feedback session 1 week later. Participants were guided by a member of the research team in individual walkthrough sessions on how to interact with the prototype. Youth and caregivers completed separate walkthroughs unless they requested to complete them together. Following their walkthroughs, participants received a password-protected link (to which only the research team had access) to their own personalized prototype that could be accessed from any smart device (eg, laptop, phone, and tablet) and used independently throughout the remainder of the study. Participants had 1 week to interact with the prototype and received US $25 in Amazon electronic gift cards. After the guided walkthrough, participants were given minimal instruction for the trial period, allowing interaction with the prototype to be self-directed.

Following the 1-week period of independent interaction with the prototype, researchers conducted a user feedback session to evaluate participants’ experiences using the prototype and how it supported self-reflection. These semistructured interviews were conducted virtually via Zoom [16] and lasted approximately 45-60 minutes. The research team conducted the youth and caregiver interviews separately unless there was a specific request to complete the interview together. Participants were asked open-ended questions about the kidney function component to probe about what they liked or disliked about the component, how they used it to reflect on their journey, and what they learned (Figure S2 in Multimedia Appendix 1). Feedback from participants on interim findings was not incorporated into the study methodology. Participants received US $25 in Amazon electronic gift cards for participating in the interview.

### Analysis

#### Overview

All sessions were audio and video recorded and later submitted to a HIPAA (Health Insurance Portability and Accountability Act)–compliant professional transcription service to be transcribed verbatim. The research team completed the analysis of both studies using MAXQDA analysis software [19].

#### Study 1 Analysis

Using content analysis [20], we analyzed deidentified design session transcripts along with participants’ drawings created during the design sessions. In total, 8 transcripts were analyzed from 8 design sessions. A total of 4 members of the research team took part in iterative rounds of transcript analysis. In the first round of analysis, each member independently analyzed and coded 1 design session transcript. The research team met after the first round of independent analysis to discuss emergent codes. After the initial round of analysis, the research team completed 3 more rounds of analysis, during which they met frequently over 1 month to discuss newly emerging codes, categorize codes based on their meanings, and resolve any analytical discrepancies or interpretations [21]. After analyzing all design session transcripts, the researchers met 3 more times to cluster and develop themes based on the codes through inductive analysis [21].

#### Study 2 Analysis

Data analysis for study 2 was conducted in two parts: (1) thematic analysis [21] and (2) affinity diagramming [22-24]. In part 1, a total of 2 researchers developed a codebook (Figure S3 in Multimedia Appendix 1) from the research questions and assessment of the My Kidney TREK app prototype. This was then iteratively refined through an additional inductive analysis of 2-3 transcripts with the rest of the team. Further, 3 rounds of coding were conducted over 3 weeks. In rounds 1 and 2, pairs of researchers independently coded 2 to 3 transcripts using the codebook and flagged emergent themes. The team then met to resolve discrepancies and revise the codebook. In round 3, a total of 4 members each coded a new transcript to validate the updated codebook. Finally, the main researcher reviewed all transcripts to ensure consistent application of codes.

After identifying relevant quotes in part 1 of the analysis, the research team performed an inductive analysis via affinity diagramming [22-24] using FigJam [25] in part 2. The research team met to sort the quotes into emergent clusters, which were discussed and refined.

### Ethical Considerations

Ethical approval for both studies was obtained from the Seattle Children’s Institutional Review Boards (STUDY00004468 and STUDY00002642). Participants in both studies received information about the study and provided informed consent and assent before study procedures were initiated. To ensure participant privacy and confidentiality, all data were deidentified and stored on secure, password-protected servers, with access restricted to the research team. All youth and caregiver participants received a US $25 Amazon electronic gift card upon the completion of each study activity, including design sessions, walkthroughs, and interviews.

## Results

### Overview

Despite study 1 and study 2 being iterative and progressive, the results indicate overlapping themes and concepts. Study 1 helped identify initial themes, while study 2 tested a prototype developed in response to those findings and further validated key themes from study 1. The findings section is organized based on these overlapping themes, in which themes 1 and 2 present results from both studies and theme 3 presents results from study 2 only (Textbox 2).

Participants are labeled by study number, role (youth or caregiver participant), and participant number—for example, S1Y1/S1C1 for Youth 1 and Caregiver 1 from Study 1, and S2Y1/S2C1 for Youth 1 and Caregiver 1 in Study 2.

**Themes from studies 1 and 2.**

**Theme 1: patient and caregiver challenges in understanding kidney function results**

- Study 1 findings: challenges in interpreting kidney function data
- Study 2 findings: reinforcing and validating information gaps

**Theme 2: emotional dimensions of kidney function**

- Study 1 findings: identifying emotional ties to kidney function
- Study 2 findings: emotional engagement with the kidney bean visual

**Theme 3: making kidney function results more accessible, understandable, and actionable**

- Study 1 findings: using longitudinal data to track kidney health
- Study 2 findings: improving information accessibility through layout and design

### Participant Characteristics

#### Study 1

Author JD recruited a total of 30 participants: 14 pediatric kidney transplant recipients and 16 caregivers for study 1 (Table 1). The median age of youth participants was 15 (range 10-21) years. A total of 51 youth and caregivers were contacted; 21 participants were uninterested or had time constraints. A total of 6 participants (3 youth and 3 caregivers) provided consent and later withdrew because of time constraints. Further, 23 participants completed the study in English, while the remaining 7 completed the study in Spanish. Most patients (12/14, 86%) reported that it was their first kidney transplant and that they had received dialysis before kidney transplantation. A total of 6 youth and caregiver participants (3 youth-caregiver pairs) from this study also participated in study 2 (institutional review board approval was obtained to include patients from study 1 in study 2).

**Table 1:** Study 1 and study 2 participant demographics.

Characteristic			Study 1, n (%)				Study 2, n (%)		
			Youth (n=14)^a^		Caregiver (n=16)		Youth (n=13)^b^		Caregiver (n=10)
Age (years)									
	10-17	12 (86)		0 (0)		9 (69)		0 (0)	
	18-21	2 (14)		0 (0)		4 (31)		0 (0)	
	>22	0 (0)		14 (88)		0 (0)		8 (80)	
	Not disclosed	0 (0)		2 (12)		0 (0)		2 (20)	
Gender identity									
	Female	5 (39)		15 (94)		6 (46)		9 (90)	
	Male	8 (61)		1 (6)		5 (38)		1 (10)	
	Nonbinary	0 (0)		0 (0)		1 (6)		0 (0)	
	Gender fluid	0 (0)		0 (0)		1 (6)		0 (0)	
Race^c^									
	Asian/Pacific Islander	2 (16)		2 (14)		3 (23)		3 (30)	
	Black or African American	1 (8)		0 (0)		2 (15)		0 (0)	
	White	6 (50)		9 (64)		8 (62)		6 (60)	
	Other	4 (33)		5 (36)		1 (6)		1 (10)	
	Not disclosed	1 (7)		0 (0)		0 (0)		1 (10)	
Ethnicity^c^									
	Hispanic and/or Latino	4 (29)		5 (31)		1 (7)		1 (10)	
	Non-Hispanic or Latino	8 (57)		11 (68)		12 (93)		9 (90)	
	Not disclosed	2 (14)		0 (0)		0 (0)		0 (0)	

^a^Among study 1 youth participants (n=14), median years since transplant was 2 years (range 0.5-14.5 years), and 9 participants (69%) received a deceased donor transplant.

^b^Among study 2 youth participants (n=13), median years since transplant was 5 years (range 0.5-14 years), and 8 participants (62%) received a deceased donor transplant.

^c^Participants could select more than 1.

#### Study 2

Authors JD and CN recruited a total of 23 participants: 13 pediatric kidney transplant recipients and 10 caregivers (Table 1). The median age of youth participants was 16 (range 13-21) years. A total of 54 youth and their caregivers were contacted; 35 participants were uninterested or had time constraints. A total of 10 youth and caregivers provided consent and later withdrew because of time constraints. All participants completed the study in English. All patients reported that it was their first kidney transplant, and most patients (10/13, 77%) reported receiving dialysis before kidney transplant.

Data collection continued until thematic saturation was achieved, with a total sample size of n=30 for study 1 and n=23 for study 2. Although the literature suggests saturation is often reached within 9-17 interviews, we engaged a larger sample of 30 participants to ensure breadth. Recruitment concluded only when concurrent data analysis confirmed that no novel themes were emerging.

### Theme 1: Patient and Caregiver Challenges in Understanding Kidney Function

#### Study 1 Findings: Challenges in Interpreting Kidney Function Data

Initially, our first 2 groups of participants (n=16) were asked to create kidney function visuals in the form of line graphs. A total of 62.5% (5/8) of caregiver participants drew and described their graphs successfully in response to the first prompt. The caregivers who did not have fully correct graphs had minor incorrect components to their drawings (S1C1, S1C5, and S1C8). Some common mistakes found in caregivers’ graphs that were semicorrect included incorrect creatinine increments and unclear graph points and line placement. Only a handful of youth participants created correct drawings and descriptions (S1Y10, S1Y5, and S1Y2), while others produced semicorrect drawings (S1Y7 and S1Y9) or incorrect drawings (S1Y1, S1Y6, and S1Y8). Some common mistakes found in youths’ graphs that were either semicorrect or incorrect included being unable to draw their creatinine results because of a lack of understanding about creatinine, incorrect creatinine increments, and incorrect placement of graph points and lines. Importantly, the correctness of the drawings was not correlated with age.

When creating their drawings for the line graph task, youth and caregivers consistently described the difficulty of understanding their laboratory results and related medical terminology. This struggle with graph literacy and terminology was reflected in the creation and descriptions of participants’ drawings. For example, S1Y1, aged 12 years, described how they relied on their caregiver to understand their creatinine values, which led to a lack of their own understanding when creating their drawings.

I think it means this [referring to graph] because I feel like grammie’s [creatinine levels] is similar to mine, even though I don’t know her levels so I just kind of guess. About 10 to 8, that seems normal to me.

S1Y1, aged 12 years

Several caregivers also discussed how their trust in the clinical care teams could influence families’ understanding and acceptance of kidney function results:

I think it really comes down to, do you have the trust in your physician to interpret those results? Because we don't have the background to interpret these results. We say, oh, 1.2, 1.3, 1.4. We know that it's with creatinine and each point is more significant than maybe in another reading for instance. But is it our job to keep track of the numbers or is it our faith in our medical team to interpret the numbers correctly? I think it's a matter of trust. Some people might not trust the results.

S1C3

Additionally, several caregivers expressed self-consciousness about not fully understanding the laboratory values and their ability to reproduce them on a graph. For example, S1C8 expressed embarrassment several times during the presentation of her graph:

You guys are all really smart and I'm clearly very ignorant. [...] And so I didn't really, I still have a hard time understanding the numbers. I just know that when Dr. X, when we were seeing her, she would tell me the numbers and they were too high. [...] I made a mistake and backwards, but I'm also probably a little dyslexic and I'm just not very math oriented. So this one, I'm embarrassed about, but yeah. I don't understand the numbers. I never have been able to.

S1C8

These sentiments revealed a broader theme: both youth and caregivers faced challenges interpreting laboratory results and medical terminology, which directly influenced how they engaged with the task of representing their kidney function on a line graph.

#### Study 2 Findings: Reinforcing and Validating Information Gaps

Reinforcing the findings from study 1, many participants expressed that they had difficulties understanding laboratory values presented in the clinic. For example, S2Y1 shared that they had an especially challenging time understanding laboratory results, tending to nod along when presented:

[...] I barely understand what those mean. Every time they pull out the lab result sheet at the doctor's office, I'm just sitting there nodding because I don't-- I understand what some of the words mean, but not when put together in that order.

S2Y1, aged 16 years

Caregivers like S2C9 expressed that they tended to avoid looking at laboratory values and numbers and focused more on actionable items. Additionally, retrospectively reflecting on their participation in study 1, they also expressed self-consciousness about their lack of knowledge of kidney function laboratory values.

When we were still seeing Dr. X, they would say, “Her creatinine is--” I don't even think she'd give me-- if she gave me a number, it would go in one ear and out the other. I'm just like, “Tell me what I got to do to make her better. Tell me what I got to do to make [inaudible],” because it didn't mean anything to me. And with S2Y9, there were no numbers. So I guess I've never-- and I was kind of embarrassed when we did the last study with the other families how well the mothers knew all about that. And that's something I, I don't know if I just avoid it out of fear, maybe, to not know. Do you know what I mean? It's just something-- but it is good for me to see that, especially going from 0.1 to 0.7. That's a huge, huge difference.

S2C9

#### Summary—Persistent Barriers to Understanding Kidney Function

Our findings indicate that youth patients and their caregivers continue to experience information gaps in (1) their overall understanding of general kidney function clinical content and (2) their ability to interpret and contextualize clinical data as it is currently presented (eg, graphs).

### Theme 2: Emotional Dimension of Kidney Function

#### Study 1 Findings: Identifying Emotional Ties to Kidney Function

**Figure 4:**
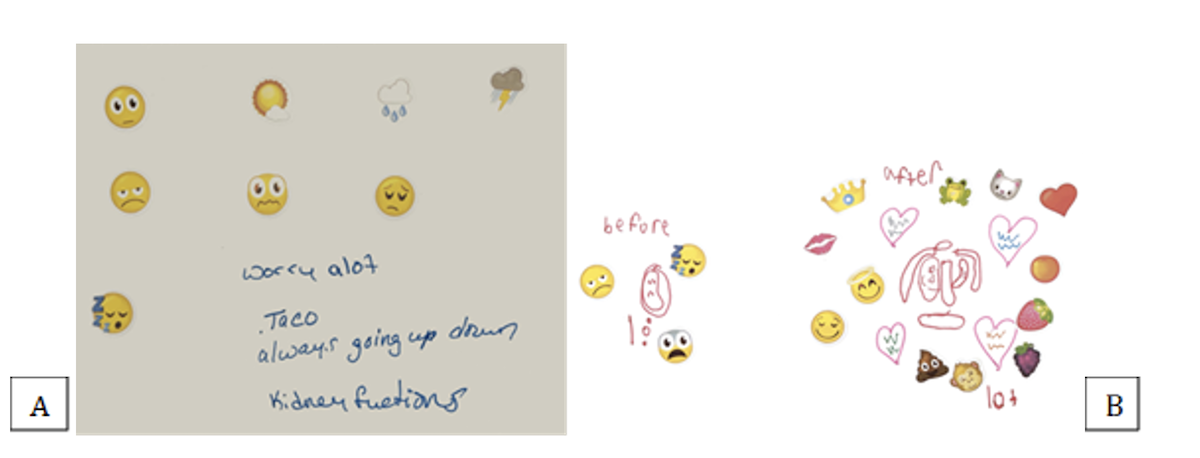
Kidney function drawings from (A) S1C11 and (B) S1Y11.

Adjusting our prompt, we asked the remaining study 1 participants (n=14) to draw any image, figure, or visual they preferred to represent their kidney function (Table 1). With this new prompt, youth and caregiver participants created and described visuals that were unexpected in several ways. One notable finding was the recurring theme of connecting emotions to kidney function in their drawings and descriptions (Table 2; Textbox 2). For instance, one caregiver chose to use the stickers we provided to express all of the different emotions that she had felt about her child’s kidney function (Figure 4A).

Because I'm still in the worry part of S1Y11's transplant, because they are still up and down with her medications...So it's just really with her since transplant, I've just been worried that she's not going to keep the new kidney, because it's just been worry after worry after worry, when she gets her blood work.

S1C11

Even though they were in different Zoom rooms, S1Y11 (Figure 4B) similarly used her emojis to represent her kidney function and how it made her feel physically and emotionally:

This is my... Before it was a 1% and I was scared that I wasn't going to get a kidney and I was sleepy and tired and it's at 1% and this is me after, happy and the fruits are for healthy and the hearts are for...[Things in the hearts] is all the stuff that I need to keep healthy like my medicine, and my stuff I need to keep healthy like fruits, lots of fruits, some vegetables because I only like some vegetables and yeah.

S1Y11, aged 14 years

Additionally, 1 notable observation is that after changing the prompt to allow for drawings of any kind to represent their kidney function over time, we found that no youth or caregiver participants chose to draw their kidney function in the form of a graph, even though they could do so.

**Figure 5:**
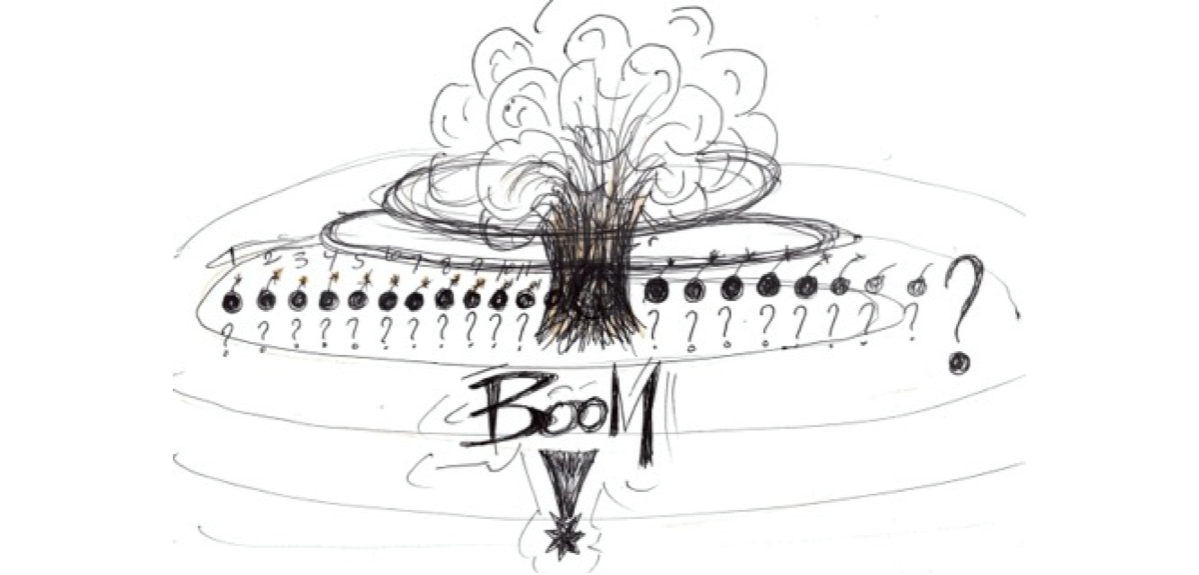
Kidney function drawing from S1C12.

For example, S1C12’s drawing (Figure 5) used the analogy of a bomb to represent how their family felt after the initial diagnosis of needing a kidney transplant through to today.

And then the question marks are we didn't know anything about what was going on until boom, we found out he needed a kidney transplant, and it just devastated our life, his life and it's like somebody dropped a bomb on us…And then still got bombs on the other side with fuses lit, because we don't know how this new kidney's going to do. And that's why the question marks.

S1C12

Each of these examples shows how participants gravitated toward creating visuals that depicted the emotions connected with their changing kidney function, rather than describing the change in kidney function through a graph. As youth and caregivers presented their drawings about the emotions tied to their kidney function, other participants often responded with similar sentiments and feelings, building rapport between participants as well as highlighting the importance and stress that comes along with doing laboratory results.

For example, S1C13 asked a follow-up question to another caregiver about their daughter’s condition and expressed similar sentiments and gratitude:

You mentioned that your daughter haven't been doing well. May I ask, is it because of her condition, or any type of rejection, notification, or any sign of rejection that's making your daughter not feel well? [After the other caregiver’s reply] Yeah, of course. Yeah, we're all going through similar... Not every situation is the same, but yeah. And I thank you for sharing that with us.

S1C13

The findings from study 1 revealed that participants consistently chose to express the emotional impact of kidney function over time, rather than focusing on clinical metrics or drawing graphs. This not only highlighted how emotion is deeply tied to understanding kidney function information but also fostered moments of empathy and connection among participants.

**Table 2:** Examples of correct, semicorrect, and incorrect caregiver and youth drawings and descriptions.

Categorization and example drawings		Quotes
Correct		
	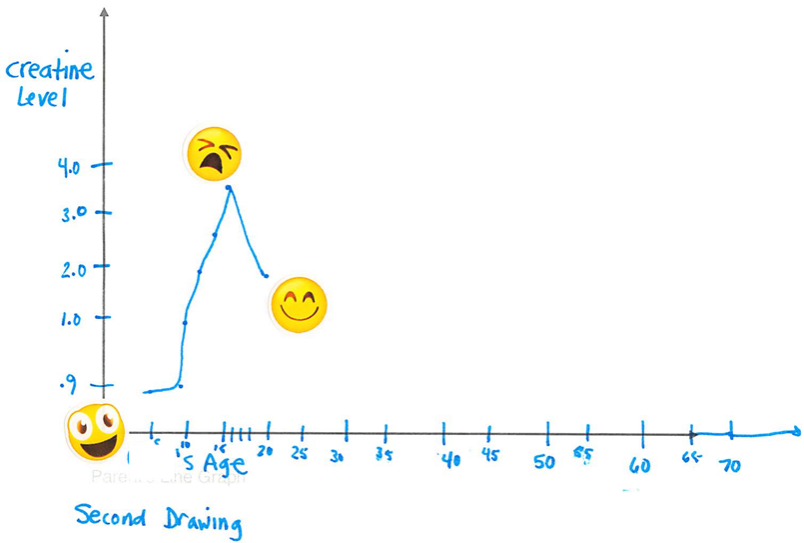	“So his creatine number looks good until he was age 12. Then it started going up at all our visits for over the last two years and then they said dialysis. Then they said, right here, it's time for transplant. Then we get the transplant, and it goes down to a more normal level, which varies for everybody...so I put him there. He's like 1.1, 1.2, but as he gets older, it'll start creeping up just like all of us and his will go faster though, because it's someone else's kidney, so it's not his own. So it'll have more challenges against it.” [S1C9]
	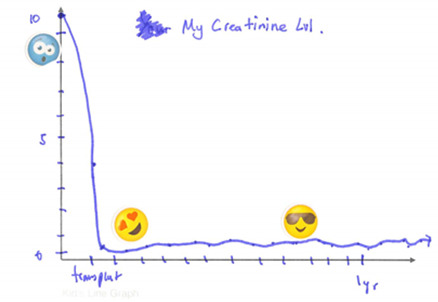	“Pre-transplant, my creatinine was very high, at 10 or something. And then transplant came all the way down to about 1.0 and then it's been up and down around the 1.0 area for last year-ish. So that was mine...The first one is surprised face because it's super high compared to now. And then this one, the heart, it went all the way down so I was like, wow, that's crazy. And then here where it's cruising along, and I thought that was cool” [S1Y5, aged 15 years]
Semicorrect		
	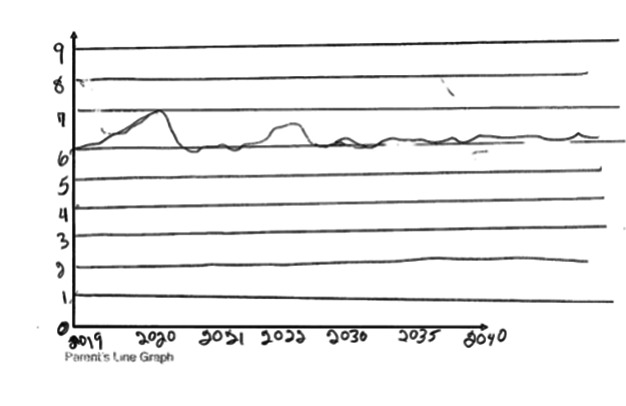	“She started out high and she really bounced this around a lot. So, she's up and down, and actually she's still an up and down. That's where she goes up and down” [S1C1]
	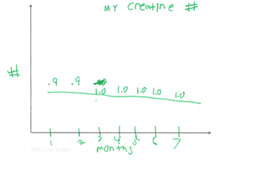	“And then here's mine for seven months. It was at 0.9 and then it went up to 1.0” [S1Y9, aged 15 years]
Incorrect		
	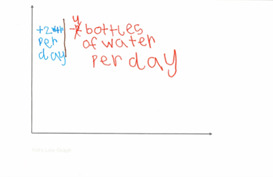	“So I am supposed to take six bottles per day. But I don't know, I only drink like two bottles of water per day, but I don't like four more bottles of all. That's all I can explain” [S1Y6, aged 11 years]
	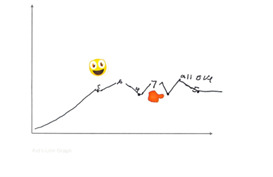	“Mine are always 5 to 6, sometimes 7. It’s very rare to have an 8.” [S1Y1, aged 12 years]

#### Study 2 Findings: Emotional Engagement With the Kidney Bean Visual

##### Overview

In response to the kidney bean visual, which the researchers developed based on insights from study 1, we found that participants identified and engaged with the emotions displayed in the visual.

##### Kidney Emotion as a Mirror of Their Own Emotions

The overwhelming majority of both youth and caregiver respondents appreciated the personification and emotional expressions of the kidney bean visual, finding the associated colors both simple and reflective of their own emotions.

The face was definitely-- it takes a kidney that nobody ever sees, hides in your body. It makes it-- like we talked about. I've talked about it for years. I was like, “Your kidney is a raisin. Go and drink some water.” Go plump it up. So it was funny to have just a face on it. And I think that as much as a 14-year-old doesn't want to admit it, I think that looking at a face is a big difference from just thinking intangibles. It's in my body.

S2C8

And I like the fact that the art changes, too. Yeah. [...] It's more like a positive thing. And it makes me feel like, “Oh, I'm happy. He's happy.

S2Y12, aged 21 years

For caregivers, resonating with the emotions tied to the kidney bean prompted reflection on their own hopes for their child’s kidney journey.

I guess, yeah, it's kind of encouraging to see how the kidney is happy in a similar way. I mean, I think this is great for a child to see, yeah, to show them how far they've come, for sure. For Y09, yes. Yeah. I mean, it's encouraging even for me to see it. And I [just want this?] kidney to last. I mean, three years. It's already flown by.

S2C9

##### Kidney Emotion as a Motivator

Many respondents shared that seeing their creatinine values over time, paired with the kidney bean visual, motivated them to take action in maintaining their kidney health. For example, S2Y7 explained how they think a visual can be more encouraging as it shows the individual a representation of the condition, rather than an abstract description.

I feel like it could be motivating to a lot of people like, ”Oh, my kidney's not doing good.“ And they can actually see that visual. I feel like a lot of people, they can hear, ”Oh, your kidney's not doing good.“ But sometimes they need a visual. Or sometimes, they'll show pictures of maybe a diseased kidney or whatever. It really encourages people like, ”Okay, I want to get on track. I want to keep my kidneys healthy.

S2Y7, aged 16 years

Multiple participants also expressed that seeing the emotions tied to the kidney bean reminded them to partake in healthy habits that maintained their kidney health.

It just, again, showed that I need to keep it healthy and do all my daily routines and medicines.

S2Y17, aged 13 years

I think it's motivating seeing the little kidney all happy and stuff. [...] I feel like that could be really an encouragement in that visual.

S2Y7, aged 16 years

#### Summary—Emotion as a Key Factor in Kidney Function Perception

From our findings in study 1, kidney function laboratory values, although seemingly straightforward in their intended purpose, that is, to inform clinical teams and kidney transplant families about a youth’s kidney function, are also tied together with the emotions of individuals and families. In study 2, we found that youth and caregivers resonated with the emotions and colors of the kidney bean visual, with the visual acting as a mirror of their own emotions and motivating them toward self-management.

### Theme 3: Making Kidney Function Data More Accessible, Understandable, and Actionable

#### Overview

In this section, we report on feedback received from study 2 on how the design of our kidney function component and kidney bean visual helped to bridge existing information gaps.

#### Study 2 Findings: Using Longitudinal Data to Track Kidney Health

Overall, participants appreciated the presentation of the creatinine laboratory values and stages over time, as it prompted reflection on their transplant process as a journey and reminded them that the journey was still ongoing.

I think this is great for a child to see, yeah, to show them how far they've come, for sure. For S2Y9, yes. Yeah. I mean, it's encouraging even for me to see it. And I just want this kidney to last.

S2C9

I liked the timeline of being able to see how bad my creatinine was or how abnormal it was and then being like, “Oh, it's good. It's normal.” And I like the fact that the art changes, too.

S2Y12, aged 21 years

In particular, caregivers noted that seeing the changes in creatinine over time helped them recognize how drastically their child’s health had improved while also serving as a reminder that ongoing effort was needed to prevent kidney function from declining.

I learned that her kidney transplant was amazing, that it brought it to such a drastic lower level than it was prior, but that it's progressively increasing. But I think that is one thing that we do look at the labs quite a bit. So, I have had that in the forefront of my mind is her creatinine, but I think that's really the only level I really paid great attention to.

S2C17

#### Study 2 Findings: Improving Information Accessibility Through Layout and Design

When asked what participants liked about the kidney function component, many identified the definitions explaining creatinine and CKD stages as valuable. This feature was well received with both youth and caregiver participants, with many noting that while these medical terms were used consistently in their care, they had never been officially defined. For example, S2Y7 noted the simple language used that might be particularly helpful for younger patients:

*Well, I did like that it shows the actual creatinine amount, like 0.44. [...] Iliked that it explained what that stage meant and stuff. And so the range and everything. I thought that was a really nice addition to help you understand, maybe, especially like I was saying for younger people or even people who have just recently got a transplant, helping them to understand what that means.* [S2Y7, aged 16 years]

Compared with the traditional presentation in the clinic, participants liked that the layout made information more easily accessible. S2Y12 remarked that it was nice to have a refresher on the definitions and details, especially since there is less opportunity for that during clinic visits.

I liked the layout of being able to look with more detail at everything because it is so much like data. I just had a doctor's appointment last week, and they were going through the blood work thing. They don't go through every single thing anymore, but I'm still like, “What is that again?” I have kept asking. So it's nice to be able to have the details of what each panel looks like in the app. I think that's really helpful and useful.

S2Y12, aged 21 years

Participants frequently expressed confusion about information presented in the clinic, which reinforces our previous findings on existing information gaps experienced by youth and caregivers. In contrast, they found the app’s presentation of information easier to understand and digest.

When the doctor's telling me something, sometimes they say words I don't understand and I'm confused. And sometimes they don't explain what it means. [...] Because I think I definitely understand it more than the things you see at doctors because I'm like, “Huh, what does that mean?” But on the app, I'm like, “Wait, I understand this.

S2Y14, aged 14 years

I think the whole kidney health part that I talked about earlier that I really liked, I didn't really ever get it broken down by stages, or at least I never really paid attention if they did break it down to me in stages.

S2C17

Many participants also expressed appreciation for the general attractiveness, usability, and educational aspects of the visual design. Participants also noted the design’s general appeal to a wider range of audiences, especially for those who do not have a health care background, such as younger patients.

#### Summary—Reflections and Actionable Insights From the Kidney Bean Visual and Kidney Function Component

In study 2, youth and caregivers valued seeing kidney function laboratory results displayed over time, as it allowed them to track their own or their child’s progress and reinforced the idea of kidney transplant as an ongoing journey. They also appreciated the simple, easy-to-understand design of the visual, especially in contrast to the confusion they often experienced with traditional clinical presentations of health information.

#### Insights From Studies 1 and 2

Ultimately, these findings highlight a critical design gap in the communication of CKD status. While themes 1 and 2 reveal the cognitive challenges and emotional resonance patients face when interpreting isolated laboratory values, theme 3 points to a possible design solution. From our findings, we can summarize that patients and caregivers require intuitive, longitudinal data visualizations that bridge the gap between abstract clinical metrics and the lived emotional experience of managing chronic illness while providing clear, actionable insights through accessible layouts.

## Discussion

### Overview

Our findings offer significant insights into patients’ and caregivers’ understanding of traditional health data presentation methods and inform the design of a novel visual that can help facilitate self-management through self-reflection and improve clinical interactions. To actively contribute to shared decision-making, patients and caregivers need access to relevant health information and the opportunity to discuss any emotional concerns it raises with their clinical team. Supporting these families in understanding kidney function laboratory results remains a critical area for further exploration, particularly in improving how clinical content is presented to them. Visualizations, as demonstrated by the kidney bean visual, can play a powerful role in simplifying complex data and prompting actionable insights that can empower patients in their self-management [12,13].

To this end, our findings identify that kidney transplant recipients and their caregivers often struggle to understand and respond to complex health information, such as kidney function values (eg, creatinine) limiting their ability to effectively engage in their care. Furthermore, kidney function laboratory values are more complex from the patient’s and family’s perspective, as they also closely align with their emotions. Keeping this in mind, we recognized that understanding the basic meaning of kidney function values may not be enough to interpret the information’s significance. By resonating with and understanding the meaning of the results, this not only informs patients but also allows them to act on the information. This is especially critical for AYAs with chronic illnesses, as developing the ability to interpret and act on health information is a vital part of building self-management skills [26]. Supporting this understanding early helps AYAs gradually take on more responsibility for their care, which is essential for eventual successful transition to adult health care [27].

Therefore, to engage individuals in their health, it is critical that numbers and values are presented in a way that represents meaning for the person receiving them and can easily become actionable. For AYAs with kidney transplants, laboratory values like creatinine and GFR are abstract and can be difficult to interpret, especially amidst all the new information they are receiving posttransplant. However, when these numbers are represented through familiar forms, such as everyday objects or emotions, they become more relatable and meaningful. Ultimately, filling knowledge gaps for patients and their caregivers can help not only encourage participation in decision-making but also empower communication between providers and patients.

In the following sections, we explore how the following principles guided the design of our novel visualization, the kidney bean visual, to convey complex health information to facilitate and improve patient engagement: (1) using visual analogies to facilitate understanding, (2) presenting longitudinal data for progress checking, and (3) designing visualizations to improve patient-provider communication.

### Using Visual Analogies to Facilitate Understanding

Traditionally, kidney function values (ie, creatinine values) are presented to patients and caregivers through conventional information visualizations (eg, line graphs). However, our findings from study 1 suggest that traditional line graphs did not fully meet the information needs of youth and their caregivers. This finding corresponds with prior investigations [28] that highlight patients living with a chronic condition struggle to interpret and use traditional graph visualizations. Participants had trouble understanding their health data through traditional visualizations because the visualizations did not reflect their circumstances, values, or emotions over time [28].

Instead, consistent with the literature [29], we found that health interfaces should prioritize nongraph visualizations, such as visual analogies. These types of visualizations simplify complex medical data and make abstract health concepts more concrete and relatable, especially for younger users. Visual analogies compare 2 concepts, 1 familiar and 1 unfamiliar, which allows the audience to use information to understand the unfamiliar concept through the lens of the more familiar concept [30]. Analogies can help humans construct mental models, which are an internal representation of the world and how it works, by basing them on concrete objects or familiar physical experiences [31,32]. The development of mental models is especially effective for youth, as it enables them to progressively refine and apply their models to more complex information over time [33,34].

Nongraph visualizations, specifically visual analogies, enhance patient comprehension of health information compared with text-only or graph visuals [29]. Incorporating familiar physical objects and color-coded cues in visualizations can enhance comprehension by linking what people already know with the features of a target concept, making the information more accessible and actionable [13].

The kidney bean visual was able to represent complex health information in a way that resonated with participants. It mirrored and validated their emotions, prompting reflection on their kidney function values within the context of their lived experiences. By being able to facilitate understanding, there is the potential for the kidney bean visual to align kidney function values with actionable self-management activities that can help build agency and self-efficacy. This is supported by prior work, which shows that personal visualizations can support self-reflection and better inform behavior change [35]. We observed this phenomenon in our participants’ language and feedback, with the majority noting the kidney bean visual’s motivational influences. Visualizations like these can be especially important for AYAs, as this developmental period is marked by increasing autonomy and formation of life skills, making it an important window for fostering the habits and health literacy needed to manage a chronic illness into adulthood.

The resonance participants reported with the emotion-integrated kidney bean visualization suggests that their laboratory values are not processed as a pure clinical metric but rather through a multidimensional emotional lens. This finding can be interpreted through the Cognitive Appraisal Theory, which posits that an individual’s emotional response is dictated by their primary appraisal of a stressor—in this case, interpreting a rising creatinine level as a “threat” to their kidney health [36]. By pairing abstract numerical data with recognizable facial expressions and color, the kidney bean visual is able to mirror a similar emotional state. Individuals can use this appraisal to prompt a secondary appraisal, a stage where the individual evaluates their options for coping with and responding to the “threat.” Within this framework, the emotional states reflected by the bean can motivate individuals to translate their emotional resonance into commitment and motivation focused on maintaining healthy habits. In addition, we found that pairing explanations with visualizations in a simple and attractive interface can enhance understanding. An estimated 1 in 3 AYAs have poor health literacy, impacting their understanding of health information [37]. Additionally, prior work has found that laypersons are more positively influenced by the attractiveness of a website’s design compared with medical professionals and have been found to reject high-quality content because of poor visual design and confusing displays [38]. Taken together, our results emphasize the need for an interface that presents health information visualizations along with concise explanations in a simple yet visually attractive manner.

To accompany the kidney bean visual, we incorporated multiple types of explanations, including definitions of common medical terms associated with kidney function (eg, creatinine and GFR), a list and descriptions of the 5 stages of CKD, and suggested healthy kidney habits (Figures 3A-D). Following guidelines by the Patient Safety Network on health literacy, the explanations were kept at a fourth- to sixth-grade level, used familiar language, and included relevant icons [39]. Keeping health literacy in mind, a simple yet attractive interface that incorporates links to definitions of terms, detailed explanations, and simple illustrations can help to increase patients’ ability to understand their personal health information, leading to higher engagement [40].

While patients have limited control over changes in creatinine caused by factors like immune rejection or disease relapse, the kidney bean visual offers an opportunity to engage with their health data in a more proactive way. By facilitating a deeper understanding of their clinical trajectory, the visualization empowers youth to better prepare for their clinic visits and plan for future health changes, while reinforcing the value of behaviors in their control, such as medication adherence and healthy lifestyle choices.

### Presenting Data Longitudinally to Track Progress

Previous research has demonstrated that self-tracking of personal health data over time can improve individuals’ ability to build awareness of their behaviors, reflect upon them, and motivate behavior change [41,42]. Displaying data longitudinally not only helps patients visualize their health journey over time but also helps contextualize their laboratory results, spurring engagement [43]. Presenting clinical data longitudinally allows patients to place medical values in the context of their life history, which patients have found therapeutic and providers have found informative [44]. We found that most participants liked seeing their health data over time, as it allowed them to: (1) see their progress and trends throughout their medical journey, (2) contextualize and draw connections from their laboratory results to how they were feeling at the time, and (3) prompt action on how they can improve or maintain their results. In this way, presenting data longitudinally allowed for increased information accessibility and actionability, as participants were able to visualize their treatment plan and kidney care habits alongside their medical implications at different points in time.

Participants liked the benchmarking aspect of viewing their laboratory trends over time, as it was a reminder of how much they have progressed or changed. This design direction was built from previous work [45] where we identified that visualizations should support appropriate benchmarking to assess one’s health status. Hence, presenting data in a way that compares youths’ current and previous health status can foster more realistic and empowering self-assessments. This method of data presentation facilitates youth’s reflections and recollections on both the challenges and successes that they have faced along their journey.

Reflective thinking about behavior is an established approach to motivating and sustaining change, as outlined in the Transtheoretical Model of Health Behavior Change [46]. Research suggests that reflecting on personal experiences can spark intrinsic motivation and sustain behavioral change [47]. By viewing their own longitudinal health data, participants engaged in consciousness raising—where individuals understand the potential consequences of their current or past behavior and the positive outcomes of change—which aligns with the self-evaluation process of behavioral change identified by the Transtheoretical Model of Health Behavior Change.

Additionally, presenting data longitudinally, unlike other visual frameworks, does not require information to be compartmentalized into distinct domains [44]. Instead, it allows for connections between specific events. This is useful, as it allows participants to identify the different factors and complex realities that influence their laboratory values. Being able to identify and connect these domains can help participants to recognize competing priorities in their own lives that may have impacted their kidney function. AYAs with kidney transplants must navigate maintaining their health alongside school, friendships, and greater independence, and having the ability to connect life events with changes in kidney function may foster greater self-awareness and support more informed, engaged self-management.

### Designing Visualizations to Improve Patient-Provider Communication

A core component of shared decision-making is for patients and caregivers to share their goals and perspectives with their health care team [48]. We have highlighted how visual analogies and longitudinal data presentations help youth and caregivers fill information gaps, ultimately encouraging greater participation in their health care. Therefore, visualizations should have the dual function of (1) aiding in the understanding of complex information and (2) facilitating triadic communication between all stakeholders about patient and caregiver goals. Triadic communication [49] refers to the communication between health care professionals, youth patients, and their caregivers. With chronic illness, there is an expectation for youth to assume more responsibility for their care with the goal of a gradual transition towards independent self-management. Ineffective patient-provider communication may reduce youth self-efficacy, adherence to medical treatments, and providers’ assessments of youth’s readiness to transition to adult care [50]. Active and effective triadic communication can improve patients’ health outcomes and satisfaction and alleviate distress for children and their parents.

Extensive work has shown that providers tend to dominate medical interactions and primarily discuss biomedical information like laboratory values, neglecting the emotional aspects during the clinic visit [50,51]. Missed empathic opportunities refer to the phenomenon where physicians provide little emotional support in response to emotional needs and concerns raised by patients [52]. Visualizations like the kidney bean visual may help address this gap by allowing patients and caregivers to connect their kidney function laboratory values with how they feel physically or emotionally, or with specific events in their lived experiences. By attaching personal meaning to the numbers, patients are able to better recognize the significance of the data in ways that resonate personally. This creates an opportunity for deeper dialogue in clinical encounters, where providers can gain insight into the patients’ and caregivers’ emotional concerns, opening space for empathic engagement and encouraging more active involvement from both patients and caregivers. Designing visuals that support both emotional engagement and understanding of complex information can be a promising approach to facilitating triadic communication.

### Limitations and Future Work

Our findings offer new insights into designing visualizations to convey complex health information to youth with kidney transplants and their caregivers. However, we recognize some limitations to this study. Our sample represents individuals from only 2 hospitals located in 2 large metropolitan areas in the United States and, therefore, may not represent findings from other geographic regions. The sample size is relatively small, considering the diversity and complexity of experiences among youth who have undergone kidney transplantation. The recruitment strategy using convenience sampling may have introduced selection bias, favoring highly motivated participants already interested in digital health management. Data regarding educational attainment, employment status, and health literacy levels were not collected as part of the demographic analysis, which limits our understanding of how these individual factors might have influenced engagement with the digital interface. Future research should use purposive or stratified sampling to ensure a wider range of patient motivation and literacy levels are represented.

A potential limitation is the risk of social desirability bias during the virtual interviews. We sought to mitigate this by using interviewers who were distinct from the development team. This separation was intended to reduce the perceived pressure on participants to endorse the prototype and to elicit more candid reflections on the visualization’s utility.

In addition, while it is important and meaningful to see emotions in visualizations, this study also showed that it can be difficult and overwhelming for some individuals, depending on their current condition and progress. Therefore, we recommend that these visualizations be used alongside regular clinical discussions, where health care providers who are trained in communicating sensitive health information can help patients understand and process the data in a supportive environment.

As presented, this work is heavily influenced by the specific pathophysiology and clinical trajectory of youth with CKD. While there are parallels with other data-intensive chronic illnesses, the application of these findings to other chronic diseases would need to be further explored.

Future work will involve further validation of the kidney bean visual and its presentation interface, including piloting its impact on patient outcomes and exploring integration into standardized clinical workflows. Given the potential to enhance communication among youth, caregivers, and clinical teams, future research should also involve clinicians to examine how these visualizations can complement the delivery of complex health information in clinical settings. Additionally, building on these principles, future efforts could explore the design of similar visuals for other health data beyond creatinine, offering patients and caregivers alternative ways to understand and engage with their care.

### Conclusion

Kidney function laboratory values, although seemingly straightforward in their intended purpose and use, are more complex from the patient’s and family’s perspective, as they also closely align with their emotions. This study demonstrates the value of designing visualizations that capture these complexities, which can create opportunities for emotional engagement within triadic communication and ultimately facilitate self-management. In this study, we present three guiding design principles: (1) using visual analogies, (2) presenting data longitudinally, and (3) designing visualizations to improve patient-provider communication, to better support the understanding of complex health information and enhance the ability of patients and caregivers to participate in their care. When information needs are met, opportunities arise for improved triadic communication, encouraging patient engagement, supporting self-management, and ultimately resulting in improved outcomes.

## Appendix

Multimedia Appendix 1 Supplementary figures.

## Abbreviations

AYA: adolescent and young adult
CKD: chronic kidney disease
GFR: glomerular filtration rate
HIPAA: Health Insurance Portability and Accountability Act
SCH: Seattle Children’s Hospital
TREK: Thinking, Reflecting, and Empowering for Kidney Transplant Recipients
