# Supplementary material for: Designing Emotion-Integrated Visualizations for Kidney Function Self-Management: User-Centered Design Study With Youth Transplant Recipients and Caregivers
Source: J Med Internet Res. 2026 Jun 15;28:e80481. doi: 10.2196/80481 (PMC13315996; doi:10.2196/80481)
Supplement: Multimedia Appendix 1 [file jmir_v28i1e80481_app1.docx]

**Multimedia Appendix 1. Study 1 and 2 Materials**

**Figure lists.**

**Figure S1.** Example line graph in design toolkit ………………………………….……… 2

**Figure S2.** User Feedback Session researcher question guide ……..................…..... 3

Figure S1. Example of creatinine and kidney function represented on a line graph included in participant design toolkit for Study 1
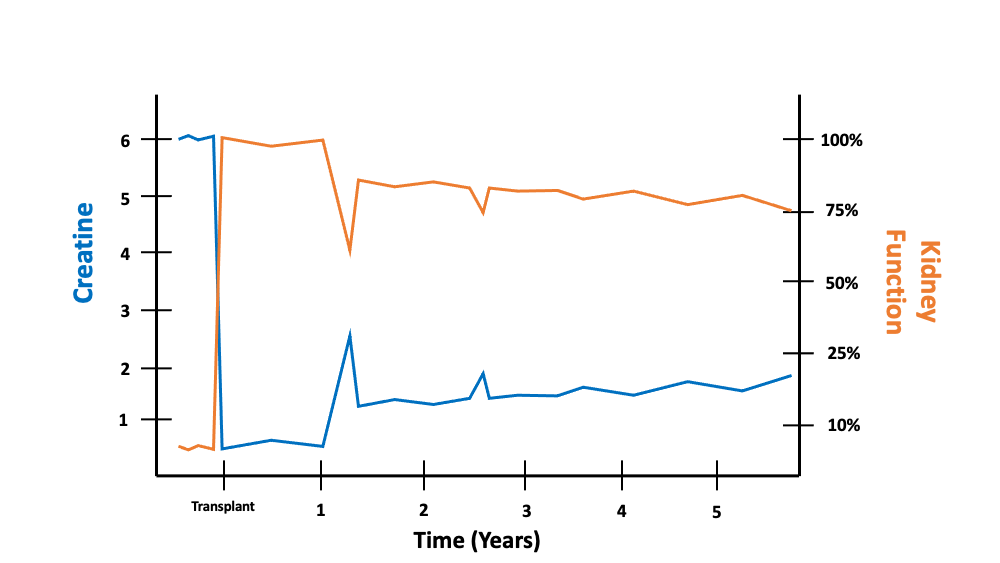


Figure S2. Question guide researchers used during Study 2 User Feedback Session


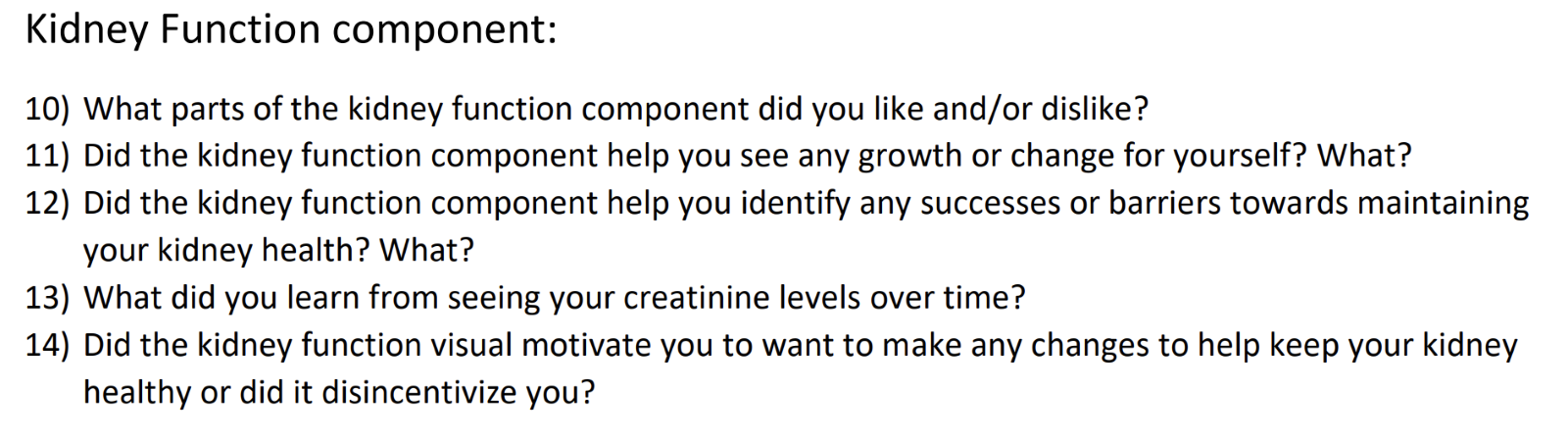


Figure S3. Summary of Study 2 analysis initial deductive codebook

| **Code System** | **Memo** |
| --- | --- |
| Code System |  |
| MY Kidney TREK - EXIT INTERVIEW CODES | These are all codes developed for analyzing the My Kidney TREK study exit interviews. |
| Reflections |  |
| Positive Reflection | Positive reflection on either self or their journey. |
| Growth/Change | Has reflected on personal growth or change throughout their journey. |
| Hypothetical Scenarios |  |
| What to Compare | Participant discusses what they would like to compare (specific components) |
| How to Compare | Participant discusses how they would like to be compared if at all |
| Mixed Thoughts on Comparison | After shown scenario explains why they have mixed thoughts on comparison or like some ideas behind and dislikes other ideas. |
| Dislikes Comparison | After shown scenario explains why they dislike the idea of comparing their or their child's personal data. |
| Likes Comparison | After shown scenario explains why they like the idea of comparing their or their child's personal data. |
| Kidney Journey Stories | Kidney journey stories specific component codes |
| KJS Design Suggestions/Ideas | KJS specific design suggestions or ideas |
| KJS Dislikes | KJS specific component dislikes |
| KJS Likes | KJS specific component likes |
| Adherence Component | Adherence specific component codes |
| Adherence Design Suggestions/Ideas | Adherence specific design suggestions or ideas |
| Adherence Dislikes | Adherence specific component dislikes |
| Adherence Likes | Adherence specific component likes |
| Kidney Function Component | Kidney function specific component codes |
| Kidney Function Design Suggestions/Ideas | Kidney function specific design suggestions or ideas |
| Kidney Function Dislikes | Kidney Function specific component dislikes |
| Kidney Function Likes | Kidney Function specific component dislikes |
| Overall Technology Probe | Any thoughts participants have about the technology probe overall. Suggest adding inductive codes as identified. |
